# A whole ecosystem approach to pear psyllid (*Cacopsylla pyri*) management in a changing climate

**DOI:** 10.1007/s10340-024-01772-3

**Published:** 2024-04-02

**Authors:** Laura A. Reeves, Michael P. D. Garratt, Michelle T. Fountain, Deepa Senapathi

**Affiliations:** 1 Centre for Agri-Environmental Research, School of Agriculture, Policy and Development, University of Reading, Reading, Berkshire, RG6 6AR UK; 2 NIAB, New Road, East Malling, Kent, ME19 6BJ UK

**Keywords:** IPM, Orchards, Natural enemies, Phenological mismatches, Trophic interactions

## Abstract

Whole ecosystem-based approaches are becoming increasingly common in pest management within agricultural systems. These strategies consider all trophic levels and abiotic processes within an ecosystem, including interactions between different factors. This review outlines a whole ecosystem approach to the integrated pest management of pear psyllid (*Cacopsylla pyri* Linnaeus) within pear (*Pyrus communis* L.) orchards, focusing on potential disruptions as a result of climate change. Pear psyllid is estimated to cost the UK pear industry £5 million per annum and has a significant economic impact on pear production globally. Pesticide resistance is well documented in psyllids, leading to many growers to rely on biological control using natural enemies during the summer months. In addition, multiple insecticides commonly used in pear psyllid control have been withdrawn from the UK and Europe, emphasising the need for alternative control methods. There is growing concern that climate change could alter trophic interactions and phenological events within agroecosystems. For example, warmer temperatures could lead to earlier pear flowering and pest emergence, as well as faster insect development rates and altered activity levels. If climate change impacts pear psyllid differently to natural enemies, then trophic mismatches could occur, impacting pest populations. This review aims to evaluate current strategies used in *C. pyri* management, discuss trophic interactions within this agroecosystem and highlight potential changes in the top-down and bottom-up control of *C. pyri* as a result of climate change. This review provides a recommended approach to pear psyllid management, identifies evidence gaps and outlines areas of future research.

## Key message

- *Cacopsylla pyri*, is the dominant UK pear pest, with an estimated cost of £5 million per annum.
- Insecticide withdrawal and resistance is driving the need for alternative control methods.
- Climate change is likely to impact this agroecosystem, potentially altering phenological events.
- A whole ecosystem approach is recommended using control methods that consider all trophic levels.

## Introduction

Historically, agricultural pest management was an oversimplified process—an insecticide or biorational compound has been applied and a reduction in the pest population expected. The observed response is often far more complex—many pest species develop resistance to pesticides, requiring the frequent development of new compounds, in this evolutionary arms race (Chattopadhyay & Banerjee 2020; Le Page 2011). Secondary pest species can also become more problematic, filling vacant niches that insecticides had emptied (Ekström & Ekbom 2011; Hill et al. 2017). Broad spectrum insecticides are a particular problem, impacting non-target organisms such as natural enemies (El-Wakeil et al. 2013) and pollinators (Connolly 2013; Kumar et al. 2018), altering the delivery of ecosystem services. Finally, weather variables can alter the persistence and mobility of insecticides (Edwards 1975; Tiryaki & Temur 2010), with light intensity, temperature and soil moisture impacting their breakdown within the environment. As these issues and challenges increased, in 1992, the United Nations Conference on Environment and Development stated that agrochemicals were the dominant form of pest control and that growers should transition to integrated pest management (IPM) (Ekström & Ekbom 2011). This management strategy would aim to maintain healthy crop growth whilst minimising disruption to agroecosystems, with focus on enhancing biological control (Moorthy & Kumar 2004). Since then, the whole ecosystem approach has become a common concept when managing agroecosystems; considering multiple trophic levels, abiotic processes and interactions between different factors (Jian & Jayas 2012; Jordan 2013).

The ecosystem approach can be applied to pear orchards, helping enhance pest management and biological control, whilst minimising synthetic chemical input. Pears are an economically important crop within the UK contributing to 2.74% of total fruit production; with a planted area of 1,477 hectares and an economic value of £15.1 million in 2022 (Defra 2023). This system has one main pest, the pear psyllid *Cacopsylla pyri*; thus, there are fewer ecological interactions to consider. Situated within the superfamily Psylloidea, there are over 4,000 described species of psyllid worldwide (Mauck et al. 2024), of these there are 24 species known species of pear psyllid (Civolani et al. 2023). These phloem feeders have a significant impact on the pear industry, nymphs produce honeydew; a sugary secretion that encourages the growth of black sooty mould on pear fruit and leaves (Daniel et al. 2005), and adult *C. pyri* are a vector of the pathogen ‘pear decline’ (*Candidatus* Phytoplasma pyri); which reduces shoot and fruit growth and can lead to tree death (Carraro et al. 2001; Kucerová et al. 2007; Süle et al. 2007). In the past, pear growers have relied on synthetic insecticides to control *C. pyri* (Civolani et al. 2023); however, over the last few decades, pear psyllid species have demonstrated resistance to multiple commonly available pesticides across the globe in particular in North America for *C. pyricola* (Harries and Burts 1965) and Europe for *C. pyri* (Atger 1979). In addition, three insecticides (thiacloprid, chlorpyrifos and spirodiclofen) commonly used for pear psyllid control have recently been withdrawn from UK use, with a fourth withdrawal planned for indoxacarb for 2024 (Hertfordshire 2023; HSE 2023), whilst abamectin and spirotetramat are in the process of being phased out in Europe (Civolani et al. 2023). Therefore, integrated pest management (IPM) has become a priority for controlling pear psylla in UK orchards (Reeves et al. 2023; Shaw et al. 2021).

*Cacopsylla pyri* have a number of natural enemies in UK pear orchards as in other parts of the world (Civolani et al. 2023; Horton et al. 2024). The anthocorid *Anthocoris nemoralis* (Fabricius) is perhaps the most documented biological control agent of *C. pyri*, whilst the European earwig *Forficula auricularia* (Linnaeus), is another key predator in orchards over the summer. Other natural enemies include: ladybird adults and larvae (Coccinellidae) (Fountain et al. 2013; Prodanović et al. 2010), lacewing larvae (Neuroptera) (DuPont & Strohm 2020; DuPont et al. 2023), spiders (Araneae) (Petráková et al. 2016), other species of anthocorid including *A. nemorum* (Sigsgaard 2010) and multiple *Orius* spp. (Vrancken et al. 2014). A few parasitoid species are also associated with pear psylla (Rieux et al. 1990; Cross et al. 1999; Jerinić-Prodanović et al. 2019), with *Trechnites insidiosus* (Crawford) commonly parasitizing nymphs in European pear orchards (Nguyen et al. 1985, Rieux et al. 1990, Armand et al. 1991, Sanchez & Ortín-Angulo 2012; Tougeron et al. 2021), although only limited records exist in the UK. With multiple natural enemy species potentially contributing to biocontrol, it is vital to consider a whole ecosystem approach when managing pear psylla populations.

Weather variables are predicted to change significantly over the next 80 years with respect to climate change; UK Climate Projections (UKCP18) predict hotter, drier summers and warmer, wetter winters across the UK (Lowe et al. 2018; Murphy et al. 2018). By 2070, summer temperatures could increase by as much as 5.1 °C under the high emissions scenario, whilst becoming up to 45% drier (MetOffice 2022), with more frequent and intense extreme weather events (MetOffice 2019). All three trophic levels (pear trees, pear psyllids and natural enemies) are sensitive to abiotic factors within agroecosystems; thus, changes in temperature, rainfall and extreme weather events could affect phenology, activity and behaviour, compromising biocontrol (Reeves et al. 2022). Climate change is likely to impact, development rates, generation times, oviposition, diapause, feeding and activity levels of insects (Karuppaiah & Sujayanad 2012), including pear psyllids and their natural enemies.

Phenological shifts are also a real concern for agroecosystems (Reeves et al. 2022) and are likely to alter pest population dynamics (Becker et al. 2015; Thomson et al. 2010). Changes in climatic conditions can lead to shifts in the timing of phenological events, resulting in phenological mismatches; where shifts in other trophic levels do not match the corresponding shift for pest species (Damien & Tougeron 2019). One example would be psyllid populations peaking earlier in the year due to earlier hatching time, but with this not coordinating with peak anthocorid or earwig emergence. In addition, climate change can lead to spatial shifts (Polce et al. 2014); altering the spatial distribution of pollinators, pests, pathogens and pear growing regions. An example of this is North America, where the pear growing region shifted from the Eastern US to the Western US during the mid-1900s. This geographic shift was largely due to difficulties in growing pear under the hot and humid summer conditions in Eastern US, which increased the risk of infection from fireblight *Erwinia amylovora* (Davis & Tufts 1941; Elkins et al. 2007).

Taking these different aspects into consideration, this review aims to (1) describe the life history of pear psyllid, (2) outline current biological and agrochemical control strategies used against them, (3) identify potential phenological and trophic mismatches that could occur as a result of climate change and (4) propose an ecosystem-based approach to build resilience into pear production systems so sustainable pest control can be maintained.

## Life history of *Cacopsylla pyri*

**Fig. 1:**
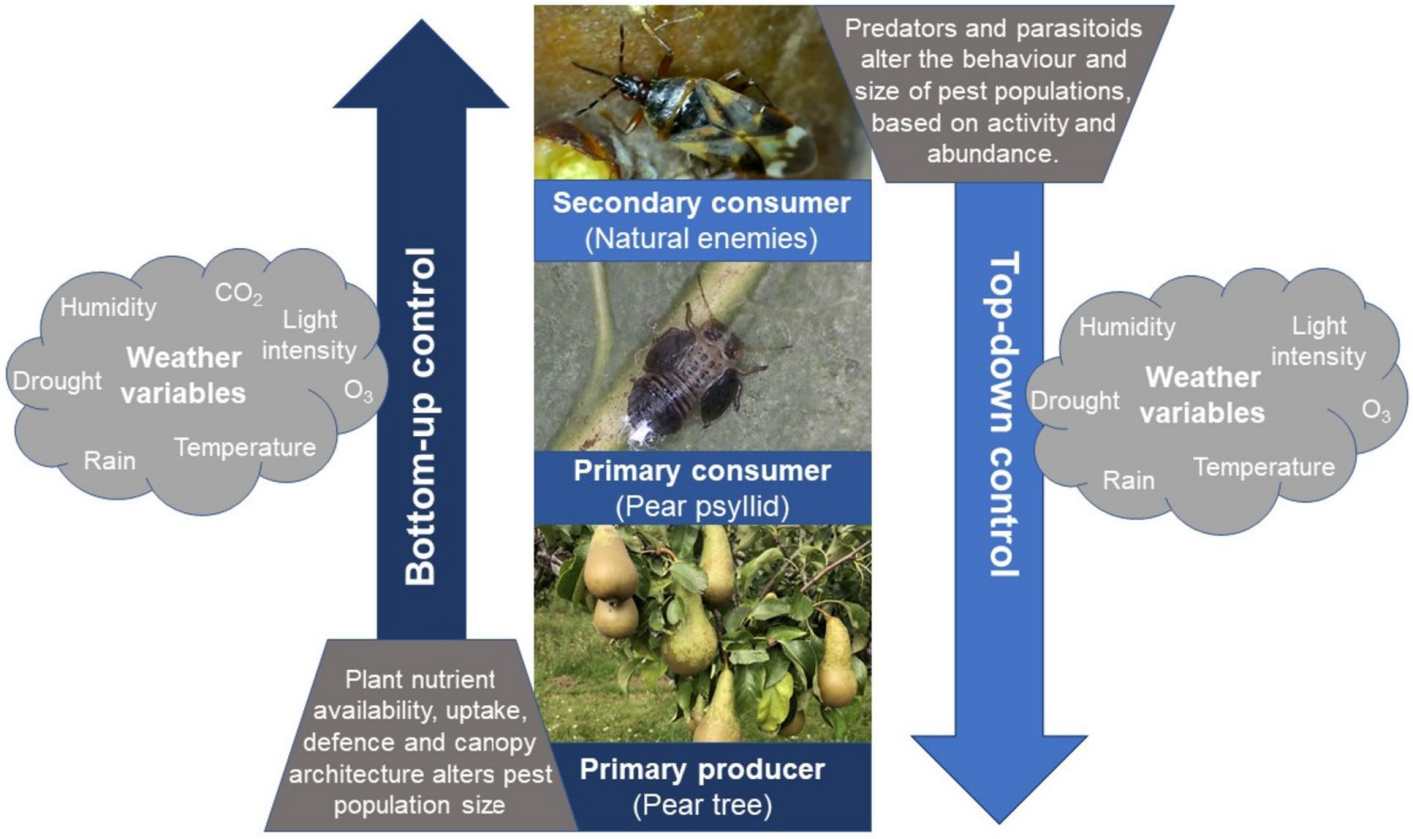
Diagram outlining bottom-up and top-down control within a pear agroecosystem and the potential interaction with weather variables, with respect to climate change. With pear trees as the primary producer (*Pyrus communis*), pear psylla (*Cacopsylla pyri*) as the primary consumer and natural enemies (including *Anthocoris nemoralis*) as secondary consumers

**Fig. 2:**
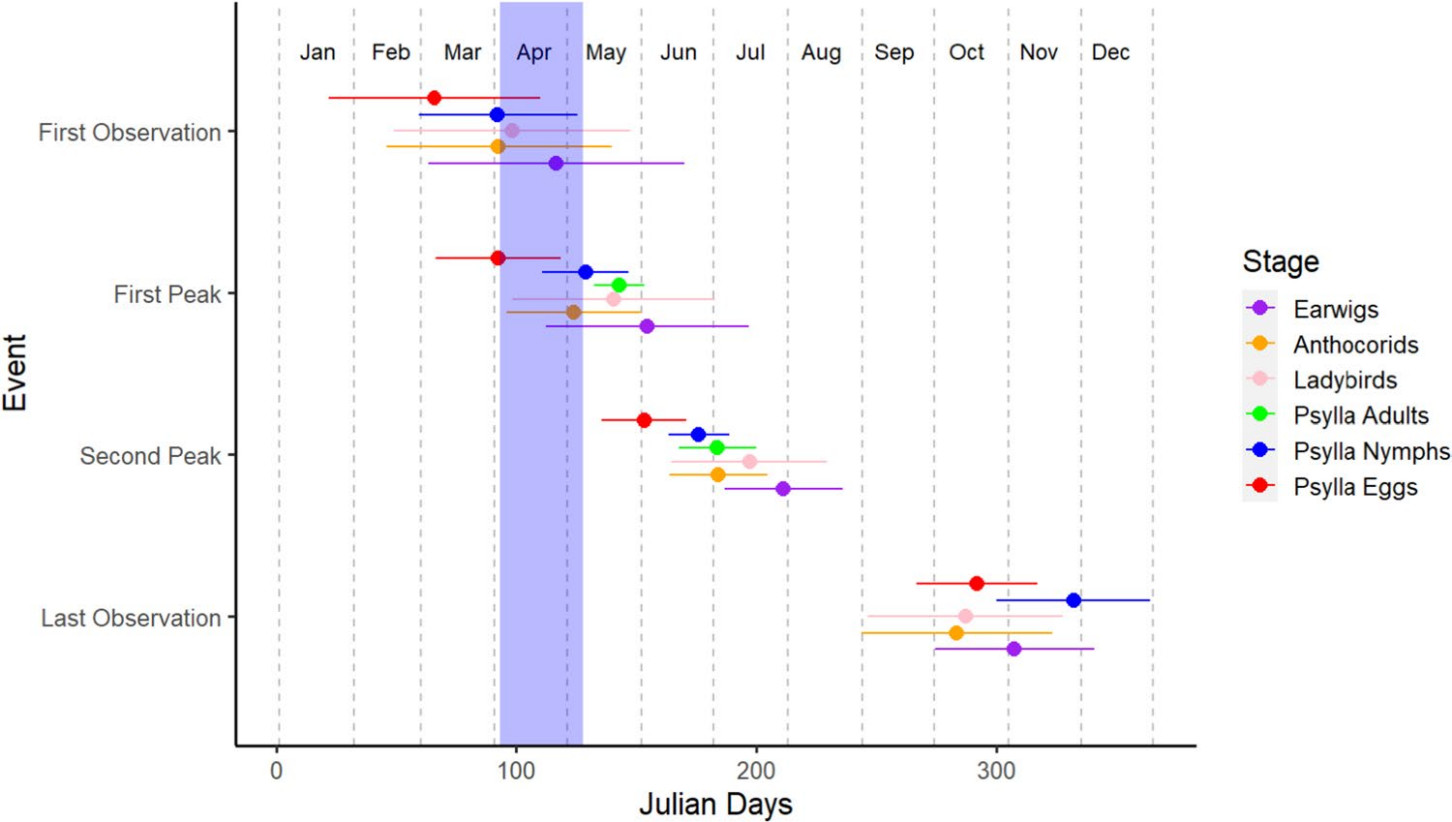
The timing of key life stages for *Cacopsylla pyri* (eggs, nymphs and adults) and its natural enemies (anthocorids, earwigs and ladybirds) in Julian days. Events include first observation in the orchard, average first peak abundance date, average second peak abundance date and last observation in the orchard. Data were collected from 17 different pear orchards in Kent, UK from 2012–2022, based on AHDB TF233 records. The dots represent the average time the event took place, lines represent standard deviation and the light blue rectangle is the average spread of flowering time for conference pear (*Pyrus communis* L.)

When taking a whole ecosystem approach to pest management, it is important to have a good overview of the target pest’s life history (Bird et al. 2009; Thomas 1999), including knowledge of oviposition, emergence time, migration, habitat preference and feeding habit. This allows for informed bottom-up and top-down control as well as providing insights into when, where and how they should be applied, to optimise the pest management strategy (Fig. 1). *Cacopsylla pyri* is currently the dominant pear psyllid species in the UK and is especially prevalent in Kent, whereas *Cacopsylla pyri*cola was previously more abundant during the 1970–1980s (Nagy et al. 2008). *Cacopsylla pyri* has two adult morphotypes (Bonnemaison & Missonnier 1955; Nguyen & Grasse 1985): a larger dark-orange black winterform (2.6–2.9 mm) with smoky-coloured wings and a smaller light-brown summerform (2.1–2.7 mm), which first appears in early May and has transparent wings. During September winterform adults begin to appear, some of which disperse from the orchard, dispersal peaks in late October or early -November, around the phenological stage of leaf fall (Civolani & Pasqualini 2003). Adults overwinter in tree bark crevices (Næss 2016), during which reproductive diapause occurs, with ovarian development happening slowly throughout the winter (Bonnemaison & Missonnier 1955; Nguyen 1975; Lyoussoufi et al. 1994; Schaub et al. 2005). By mid-late winter, female ovaries are fully developed (Schaub et al. 2005) and egg laying starts in late February to early March (Næss 2016; Oz & Erler 2021), when temperatures reach > 10 ˚C. For UK, pear orchards average first oviposition date and other key phenological events are shown in Fig. 2, based on 10 years of monitoring data.

*Cacopsylla pyri* eggs begin to hatch in early spring (Sanchez & Ortín-Angulo 2012), going from a creamy-yellow to orange when mature; the eyes are often visible prior to eclusion. Nymph emergence often coincides with bud opening, and there are five nymphal stages, each ending in a moult (Civolani et al. 2023). Early stage nymphs (1–3) are light yellow coloured, whilst older stages (4–5, hardshell nymphs) are dark-brown and larger in size, with more developed wingpads (Le Goff et al. 2021). The first peak in the pear psyllid population is seen around April–May when summerforms emerge; this is followed by a second-generation in early summer (Fig. 2). The following generations overlap throughout the summer and autumn (Civolaniet al. 2023), with an average of 3–5 generations per year (Suele et al., 2007), although generation number can be temperature dependant (Kapatos and Stratopoulou 1999).

Pear psyllids use a pierce-sucking stylet to feed on phloem sap (Civolani et al. 2011), this sap is comprised mostly of two sugars (sorbitol and sucrose) and it also contains 17 free amino acids (Le Goff et al. 2019). In order to obtain essential amino acids, psyllids consume large amounts of phloem sap, egesting a large proportion of sugars as honeydew (Le Goff et al. 2019). Nymphs egest larger quantities of honeydew than adults (Civolani et al. 2023). Honeydew can be particularly problematic in pear orchards, encouraging the growth of black sooty mould, which reduces the photosynthetic ability of leaves and reduces economic value of fruits (Daniel et al. 2005;). In addition, adults are a vector of pear decline phytoplasma (*Candidatus Phytoplasma* pyri) (Carraro et al. 2001; Suele et al., 2007); phloem sap is ingested by psyllids from an infected tree and transmitted to other pear trees via salivation into cells or tissues when feeding ( Sugio & Hogenhout 2012; Cruz et al. 2018). Pear decline can lead to reduced foliation, leaf drop and tree death, although susceptibility can depend on rootstock and cultivar (Avinent et al. 1997; Carraro et al. 2001; Çağlayan et al. 2022). Indeed, it is estimated that pear psyllid costs the UK pear industry £5 million per annum due to crop damage and control costs (AHDB 2012).

## Monitoring methods and abundance thresholds

Monitoring pear psylla is particularly important when considering the timing of control methods, as information on adult dispersal, spring oviposition and population densities, and structure is required for management decisions (Horton 1999), making it necessary to monitor pear orchards regularly throughout the year. Monitoring adults and eggs before budburst (late January onwards) is considerably important, as this is when psylla are more active in orchards and begin oviposition; thus, the application of kaolin is often necessary (Pasqualini et al. 2002). Adults can be monitored either using beat tray sampling or yellow sticky traps (Burts & Retan 1973; Horton 1999; Marcasan et al. 2022). Eggs can also be counted by inspecting the budwood using a hand lens or by taking a small sample and counting eggs under a light microscope (Horton 1999). During spring and summer, it is also important to sample *C. pyri* nymphs, as these produce large quantities of honeydew production leading to the growth of black sooty mould (DuPont et al. 2023; Nin, et al., 2012). Furthermore, under warmer temperatures adults more active and likely to fly away, so are more difficult to count via beat tray sampling (Horton 1994).

## Chemical control strategies and biorational compounds

**Fig. 3:**
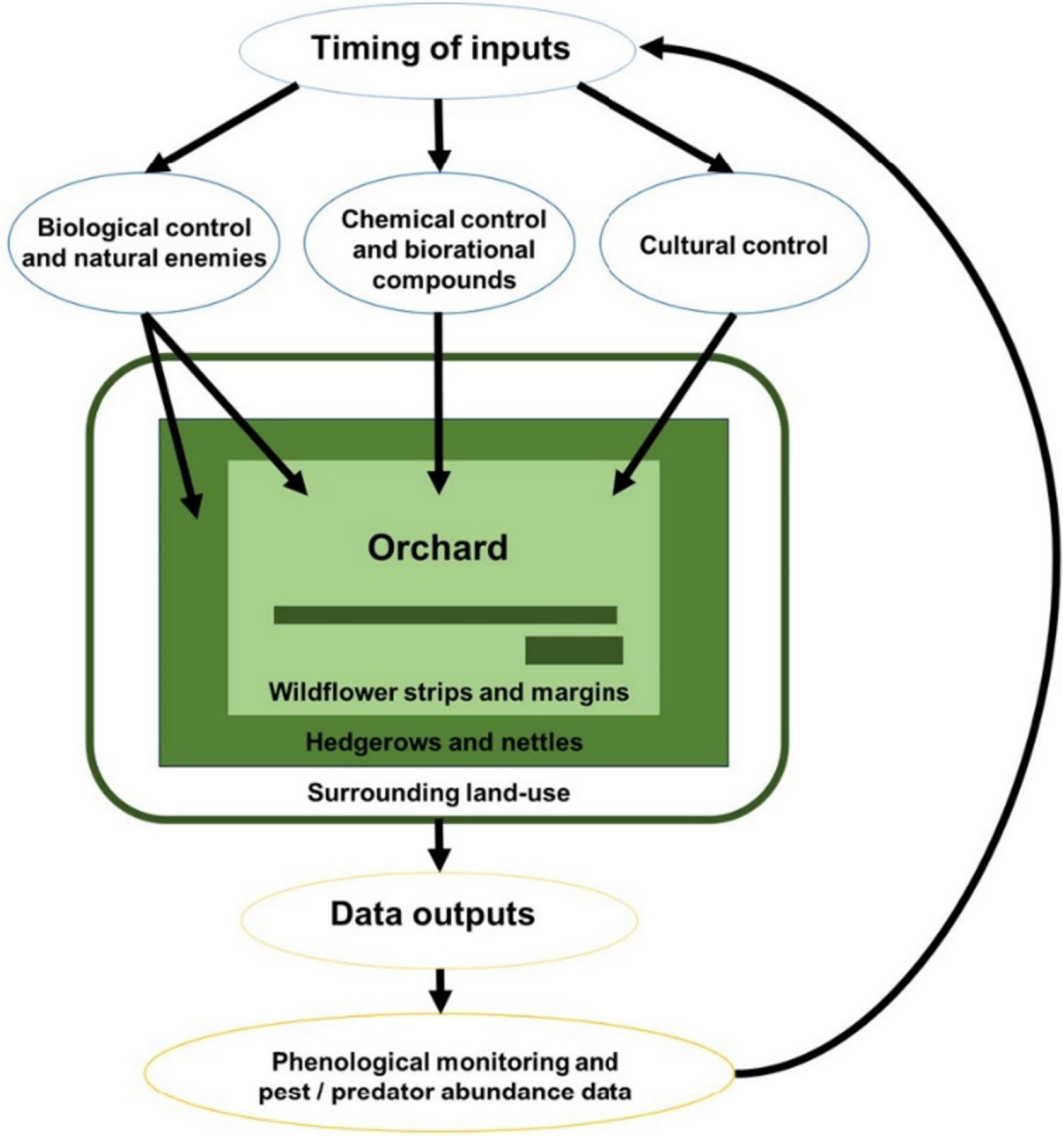
The inputs and outputs within a pear orchard that constitute pest management. Inputs include biological control, chemical sprays, biorational compounds and cultural control. Outputs are the data that growers, agronomists and researchers collect which go back into the system to optimise the timing of different control methods, maximising the control of the pest, whilst minimising damage to natural enemies, pollinators and other non-target organisms

Although, IPM focuses on minimising the use of agrochemicals, whilst conserving natural enemy populations (Wearing 1988), the application of agrochemical sprays is sometimes necessary as a last resort (Deguine et al. 2021). IPM integrates the use of chemicals in an agroecosystem by: considering spray timing (Fig. 3) and spraying when natural enemies are not yet present in orchards (Tang et al. 2010), selecting compounds that are specific to the target pest rather than broad spectrum insecticides (Zalucki et al. 2009), using biorational pesticides (pesticides made of natural products, with low environmental and mammalian risk) (Haddi et al. 2020) or biological control agents as alternatives when possible (Matthews 1999) and rotating insecticide family usage, so that pests are less likely to develop resistance (Walker et al. 2001). In this section, we will discuss the pesticides and biorational compounds commonly used in the control pear psylla (Table 1), providing an overview of how these control methods could be impacted by climate change.

**Table 1:** Biorational compounds, agrochemical sprays and biocontrol agents used within UK orchards. Based on AHDB TF233 records from 20 orchards in Kent during 2016, 2017, 2018 and 2021. Including the name of the product used with its active ingredient in brackets, the average first application date for the product, the average number of applications pear year in an orchard, a brief description of how the product targets pear psyllid and the percentage of growers surveyed that use the compound or used the compound before its ban. Compounds still approved for use in the UK are in bold, based on the description in the Health and Safety Executive (HSE) (HSE 2023) and the University of Hertfordshire Pesticide Properties DataBase (PPDB) (Hertfordshire 2023)

Product name	Average 1st application	Average no. applications	Growers using (%)	Activity	Approved/ Withdrawn
Agricolle (Natural polysaccharides)	1st Jun	1	5.00	Immobilises insect and clogs sphericles, causing rapid death through asphyxiation (broad spectrum)	Approved
AnthoPAK 500 (*Anthocoris nemoralis* adults)	4th May	2.58	35.0	A natural enemy of pear psyllid that predates on its nymphs and eggs	Approved
Batavia (Spirotetramat)	9th Jun	1	20.0	Inhibits lipid biosynthesis in phloem sucking pests (broad spectrum)	Approved until (31/07/2029)
Bittersaltz/ Epso Microtop/ Kieserite (Magnesium sulphate)	18th May	4.88	85.0	Primarily used as a fertiliser but can also help remove honeydew from leaves	Approved
Calcifert/ Lime (Calcium carbonate)	10th May	1	25.0	Primarily used as a fertiliser but can also help remove honeydew from leaves and strengthen leaf against feeding	Approved until (31/08/2024)
Calypso (Thiacloprid)	25th Mar	1.17	70.0	Disrupts the insect’s nervous system by stimulating nicotinic acetylcholine receptors (broad spectrum)	Withdrawn (31/03/2020)
Chlorpyrifos (Chlorpyrifos)	21st Mar	1	15.0	Impacts the insect’s nervous system by inhibiting the breakdown of the neurotransmitter acetylcholine (broad spectrum)	Withdrawn (01/04/2016)
Envidor (Spirodiclofen)	3rd Jul	1	65.0	Inhibits lipid biosynthesis in phloem sucking pests (broad spectrum)	Withdrawn (31/01/2022)
Explicit/ Steward (Indoxacarb)	4th Jun	1.40	40.0	Blocks insect sodium ion channels, dysregulating neuron firing (broad spectrum)	Withdrawal planned (31/10/2024)
Headland Magnesium (Magnesium)	29th May	1	15.0	Primarily used as a fertiliser but can also help remove honeydew from leaves	Approved
Headland Sulphur (Sulphur)	10th Apr	5	85.0	Primarily used as a scab and mildew treatment but can also help remove honeydew from leaves	Approved
Karamate (Mancozeb)	21st May	2.69	45.0	Primarily a fungicide but has also been shown to have insecticidal properties on pear psylla	Approved until (31/01/2024)
Mainman (Flonicamid)	10th May	1	5.0	Disrupts potassium ion channels, inhibiting the release of honeydew and saliva, leading to the cessation of feeding (specific to phloem feeders)	Approved until (31/08/2026)
Soap (Sodium hydroxide)	9th Jun	1.67	15.0	Removes honeydew from leaves	Approved
Surround (Kaolin)	30th Mar	1	25.0	A mineral-based particle film, that forms a protective barrier, repelling pests and reducing movement, feeding and oviposition	Approved (31/08/2024)
Wetcit (Alcohol Ethoxylate)	8th May	3	20.0	A wetting aid surfactant, that improves the spread and penetration of insecticides and other agrochemical sprays	Approved substance without pesticidal activity

To highlight which control methods were most common in the UK, we compiled spray records from 20 different pear orchards. Nine different agrochemical or biorational compounds were used in pear psyllid management, five involved in honeydew removal (including sulphur and magnesium sulphate for desiccation and soap to wash off honeydew), one surfactant used to enhance insecticide application and one biological control agent (Table 1). The most common insecticide used in *C. pyri* control between 2016 and 2019 was thiacloprid (product name Calypso, used in 70% of orchards), with recommendation of use before flowering. The active ingredient thiacloprid is a neonicotinoid insecticide which targets the nicotinic acetylcholine receptor and interrupts transmissions of synaptic signals, resulting in paralysis of insects (Bangels et al. 2010). Although effective in controlling the first generation of *C. pyri* (Bangels et al. 2009), the approval for the UK usage was withdrawn in early 2020 (Bellis & Suchenia 2022), due to toxicity to non-target organisms including multiple bee species (Claus et al. 2021; Orčić et al. 2022), natural enemies (Van de Veire & Tirry 2003) and soil invertebrates (De Lima e Silva et al., 2017). In total, three insecticides (thiacloprid, chlorpyrifos and spirodiclofen) commonly used for pear psyllid control have been withdrawn for the UK usage, with a fourth withdrawal planned for the active ingredient indoxacarb (Table 1). With the recent withdrawal of multiple insecticides used to target pear psylla, reliance on other insecticides and biorational compounds may become more common. Currently, spirotetramat (Batavia) is approved for use in the UK orchards (HSE 2023), it is a systemic insecticide that is translocated throughout the xylem and phloem, inhibiting lipid biosynthesis in sucking pest species (Brück et al. 2009; Nauen et al. 2008). Studies suggest that spirotetramat is particularly effective against psyllid nymphs (Civolani et al. 2015) and does not adversely impact European earwig (Shaw & Wallis 2010) or *A. nemoralis* populations when applied in orchards (Pasqualini et al., 2002), although there is some concern about its impact on predatory mites (DuPont & John Strohm 2020).

In addition, the use of the biorational compound Kaolin has become more frequent (Pasqualini et al. 2002; DuPont et al. 2021). This finely powdered clay can be sprayed onto plant surfaces, creating a non-toxic particle film (Erler & Cetin 2007). The porous white barrier can deter adult psylla from colonising orchards, reduce oviposition and impair movement via the attachment of heavy particles to the bodies of psylla (Erler & Cetin 2007; Saour et al. 2010). Pre-bloom application (February – April) of kaolin is recommended, when adult psylla are actively recolonising orchards, impact on natural enemies is minimal and spray coverage is optimal, without impacting photosynthesis as foliage is not yet present (DuPont et al. 2021). Oils are also effective biorational compounds used to suppress pear sucker during the pre-bloom stage (Civolani 2023; Emami 2023; Erler 2004a, b), interfering with colonisation of orchards and egg deposition (Pasqualini et al. 2002). One study by Pasqualini et al. (2002) found that in early spring *C. pyri*, egg numbers were 3.2 times lower on buds treated with mineral oil, compared to the untreated control. Whilst Erler (2004a, b) found that cotton seed oil, fish-liver oil, neem oil and summer oil all promoted *C. pyri* oviposition deterrence, with fish-liver oil and summer oil exhibiting 100% deterrence in winterforms over the 3-week treatment period; however, there is the issue of allergens in some oil types.

Reflective mulches have been demonstrated to suppress *C. pyricola* populations (Nottingham & Beers 2020; Nottingham et al. 2022). These are ground covers that reflect solar light into the orchard canopy (Shimoda & Honda 2013). Insects are particularly sensitive to UV light, ambient UV can promote flight behaviour (Nottingham & Beers 2020), whilst direct UV can damage eggs and nymphs (Beard 1972). Nottingham & Beers (2020) found significantly fewer first-generation (during May) pear psylla adults, eggs and nymphs in reflective-mulch treatments compared to black-mulch and no mulch treatments. However, the second-generation (June–July) of pear psylla was not supressed by reflective-mulch. This could be due to the fact that multiple natural enemy groups (important for summer psyllid control) were also reduced in the reflective mulch treatment, as UV impacts multiple insect species. Therefore, using reflective mulch during the early season may be more effective for pear psyllid control, as natural enemies are less abundant.

Pest monitoring and mating disruption through the use of pheromone lures are deployed for multiple pest species in particular Lepidoptera (Ganai et al. 2017). To date the sex pheromone of the pear psyllid species, *C. bidens* (Soroker et al. 2004) and *C. pyricola* have been identified, isolated and synthesised (Guédot et al. 2009; Yuan et al. 2021). Furthermore, there is also evidence for increased levels of the same compound in cuticular extracts of adult *C. pyri* females (Ganassi et al. 2018). Ganassi et al (2018) showed that male *C. pyri* displayed a significant preference for odours from female conspecifics and female cuticular extracts in Y-tube olfactometer assays, suggesting that a similar female-produced pheromone is likely present in *C. pyri*. Visual and acoustic signals have the potential to enhance mating disruption (Jocson 2023; Krysan & Horton 1991). *Cacopsylla pyri* have a preference for green visual cues (525 to 537-nm) (De Jorge et al. 2023), which can be used in sticky traps for psylla monitoring and control. There is also potential to supplement these traps with pheromone lures (Guédot et al. 2009; Yuan et al. 2021) to increase catch rate. Acoustic signals have an important role in psyllid mate choice (Percy et al. 2006; Liao et al. 2022); Eben et al. (2015) were first to describe the male and female acoustic signals for a pear psyllid (*C. pyri*). Jocson (2023) found that the playback of white noise and male psyllid song reduced offspring number compared to the control treatment, due to mating disruption. However, interactions between visual, acoustic and chemical signals involved in pear psyllid mate choice are under-researched.

## Biological and cultural control strategies

Natural or biological control strategies encompass bottom-up or top-down control (Fig. 1). Top-down control can be defined as a predator mediated process, when higher trophic levels influence levels below them, by altering prey behaviour or reducing pest populations through consumption of prey (Daugherty et al. 2007; Hayward et al. 2019). Top-down control is key to biological control methods used in IPM of pear sucker, either through conserving natural enemy populations, increasing recruitment of predators and parasitoids into orchards or artificially releasing biocontrol agents (Daugherty et al. 2007). The anthocorid *A. nemoralis* is the dominant predator of *C. pyri* in the UK, with the average female estimated to consume approximately 5000 psyllid eggs in its lifetime (Yanik & Ugur 2004). Adult anthocorids migrate into orchards April–May from surrounding hedgerows (Reeves et al. 2023). Eggs are laid and anthocorid populations peak mid-summer, allowing for the effective control of pear sucker (Nagy et al. 2008; Scutareanu et al. 1999). However, natural anthocorid populations do not always establish quickly enough to keep *C. pyri* populations at an economically viable level (Civolani 2012; Sigsgaard et al. 2006b). Therefore, it has become common practice in some UK orchards to mass release *A. nemoralis* (Augmentative biological control) rather than relying on enhancing natural populations alone (Conservation biological control). This review found that 35% of the orchards surveyed used AnthoPAK 500 (Table 1), a product containing 500 adult *A. nemoralis* in a dispersing material (Bioplanet 2023), available from multiple biological control companies. Sigsgaard et al (2006b) suggest between 1000 and 1500 adult *A. nemoralis* should be released hectare at 5–6 points within a pear orchard. Furthermore, timing is critical for artificial releases of *A. nemoralis*, with evidence of successful releases during early-mid May (Sigsgaard et al. 2006a).

In addition to *A. nemoralis*, many other species of natural enemy are involved in pear psyllid management (DuPont et al. 2023; Nottingham et al. 2023), among them are spiders (Araneae) (Sanchez & Ortín-Angulo 2012), European earwigs (*Forficula auricularia*) (Fountain et al. 2013) ladybird adults and larvae (Coccinellidae) which are generalist predators (Fountain et al. 2013; Prodanović et al. 2010), lacewing larvae (Neuroptera) (DuPont & John Strohm, 2020; DuPont et al. 2023) and the parasitoid *Trechnites insidiosus* (Sanchez & Ortín-Angulo 2012). European earwigs are common in pear orchards; stage four earwig nymphs are arboreal, appearing in pear trees in late spring and peaking in June, whilst adult populations peak in mid-July (Gobin et al. 2008; Moerkens et al. 2011). Earwigs are effective predators of *C. pyri* (Gobin et al. 2008; Lenfant et al. 1994), and unlike *A. nemoralis* migrations, their abundance in orchards is less dependent on *C. pyri* density. A study by Lenfant et al (1994) found that arboreal *F. auricularia* nymphs ate a daily maximum of 10 mg of psyllid prey (1000 psylla eggs), highlighting their efficiency as biological control agents. Although earwigs are omnivorous and sometimes consume plant material, damage to top-fruit is minimal (Solomon et al. 2000).

To date no biological control company rears *F. auricularia* for mass release, thus the reliance on enhancing earwig populations and providing refugia is common in top-fruit orchards (Shaw et al. 2021). One such refuge is the Wignest; a wooden shelter preloaded with a food attractant, available from the biocontrol company Russel-IPM (Russel-IPM 2023; Shaw et al. 2021). Artificial refuges can also be constructed using straws or corrugated cardboard in a bottle attached to a tree (Hansen et al. 2005; Solomon et al. 1999). Furthermore, dried cat-food is often placed in refuges as a prey supplement (Shaw et al. 2021). The benefits of using refuges in the tree canopy are that earwigs are housed arboreally and therefore more likely to forage on insects in the tree canopy when they emerge to feed at night.

Hedgerows (Nagy et al. 2008; Scutareanu et al. 1999), nettles (Shaw et al. 2021), cover crops (Horton et al. 2009) and wildflower strips (Balzan et al. 2014; Mateos-Fierro et al. 2021) can also enhance natural enemy populations, providing refuges and alternative resources for predators before they migrate or “spillover” into nearby orchards (Horton 2024). Scutareanu et al (1999) found that the first peak of adult anthocorids in pear orchards was always later than the first peak in hedgerows, indicating that anthocorids use hedgerows as refugia before migrating into orchards when psyllid populations increase. Furthermore, hawthorn was the dominant source of *A. nemoralis* for migration to psylla infested trees. This is supported by Nagy et al (2008), who found high numbers of adults on hawthorn, goat willow and stinging nettle during mid-April to May.

Surrounding land-use has also influences both pear psylla and their natural enemies (Miliczky & Horton 2005; Rendon et al. 2021; Shaltiel & Coll 2004); surrounding vegetation can act as a source or sink for pests and beneficials throughout the year, especially between growing seasons (Rendon et al. 2021). Impacts on pest populations can be dependent on land-use type (Karp et al. 2018), land-use diversity (Veres et al. 2013), size of surrounding land area and distance from orchard (Miliczky & Horton 2005). Rendon et al (2021) found that pear orchards surrounded by high cherry orchard cover had a negative correlation with predator abundance and higher pear psylla abundance, this could indicate that cherry is a less important source of pear psyllid predators, compared to more heterogeneous landscapes.

Bottom-up control is important for IPM of pear psylla (Daugherty et al. 2007); this is a resource mediated process (Fig. 1), where plant quality and chemical defences can influence pest populations, impacting prey abundance for predators (Han et al. 2022). Nutrient inputs have a significant impact on plant quality but can also influence pest populations (Daugherty et al. 2007; Kocourek et al. 2021); nitrogen is a limiting factor in the diets of pear psylla, as there are low levels of amino acids in phloem sap (Le Goff et al. 2019); thus, the addition of nitrogen fertiliser can remove this limiting factor and increase the amount of nutritious new foliage for nymphs and adults to feed upon (Daugherty et al. 2007; McMullen & Jong 1977). Daugherty et al (2007) found that pear trees given a high nitrogen fertiliser treatment had a significantly lower C:N ratio (higher N) in leaf samples and a significantly higher abundance of pear psylla (eggs, nymphs and adults) in mid-July, compared to low N treatments. Thus, controlling fertiliser inputs to provide just enough for fruit set (Civolani 2012; Daugherty et al. 2007; Nin et al. 2012), alongside an effective pruning method (Francke et al. 2022; Fuog 1983), is important for IPM of pear psylla. Franke et al. (2022), recommends removing watersprouts (soft vertical shoots) between late May—early June in a period of low rainfall, as an effective method of controlling psylla populations and reducing tree vigour.

Host resistance is another method of minimising damage from pear psylla populations (Ninet al., 2012; Shaltiel‐Harpaz et al., 2014). Resistant phenotypes may exhibit antixenosis (pest deterrence) (Bell & Puterka 2003; Nin et al. 2012), or antibiosis (when plants have a deleterious effect on a pest) (Peterson et al. 2017), reducing a pest’s longevity, development rate or reproductive potential (Shaltiel‐Harpaz et al., 2014). A resistant cultivar can be selected by monitoring pest oviposition rates, pest mortality, feeding and development rates and nymphal weight gain (Bell & Puterka 2003; Berrada et al. 1995; Pasqualini et al. 2006). Based on the UK horticulture statistics, Conference pear (*Pyrus communis* cv. Conference) is the most common pear cultivar in the UK, accounting for 84.01% of total planted area of pears (Defra 2023). However, cv. Conference alongside other common UK pear cultivars including Comice, Concorde and Williams Bon Chretien are susceptible to *C. pyri* (Berrada et al. 1995; Nin & Bellini 2000). Hybridisation of susceptible species with resistant ones can be successful in increasing host plant resistance (Harris 1973; Nin et al. 2018). Multiple intraspecific pear hybrids demonstrate high resistance to *C. pyri* infestations (Robert & Raimbault 2004). However, the fruit quality of hybrids is often a concern within breeding programmes (Ninet al., 2012; Robert & Raimbault 2004), highlighting the need to consider resistance, yield and fruit quality during cultivar selection. This challenge can be solved with the DNA marker (Dondini et al. 2015; Montanari et al. 2015).

A more recent approach to bottom-up control is through activating plant defence pathways using plant defence elicitors (PDEs) (Orpet et al. 2021; Saour et al. 2010; Civolani et al. 2022). One example is the Harpin 44-kDa protein, encoded by the hrpN gene from the bacterium *Erwinia amylovora*, which activates the salicylic acid, ethylene and jasmonic response pathways, stimulating plant growth and defence (Saour et al. 2010). A study by Saour et al. (2010) found numbers of *C. pyri* nymphs was significantly lower in the Harpin treatment compared to the untreated control and had a higher fruit load. However, other studies have only found partial or variable pear psyllid suppression using PDEs (Cooper & Horton 2017; Orpet et al. 2021; Civolani et al. 2022), suggesting that PDEs should be used alongside other control methods. Weather dependence of control methods is also important in pear psyllid management (Civolani 2012). Rainfall is perhaps the most disruptive to chemical and biorational methods, with the ability to wash insecticides and particle films off foliage and plant material (Erler & Cetin 2007) and disrupt pheromones or other chemical cues (Johnston et al. 2022). Whilst temperature may have more of an impact on biological control methods impacting feeding, development and oviposition of natural enemies. Potential disruptions to IPM with respect to weather variables are considered throughout this review.

## Phenological shifts and mismatches within agroecosystems

Multiple studies suggest that temperature significantly influences budburst and flowering phenology (Amano et al. 2010; Auffret 2021; Fitter & Fitter 2002). Fitter & Fitter (2002) highlights that flowering time has advanced rapidly in the UK over the past few decades; with first flowering time averaging 4.5 days earlier compared with the previous 40 years. Whilst Amano et al (2010) predicted first flowering to be an average of 5.0 days earlier for every 1 °C of warming, with February—April temperatures being most closely correlated to flowering phenology. This phenological advancement depending on temperature has been noted in several tree-fruit species including apples (Guédon & Legave 2008), plums (Cosmulescu et al. 2010), cherry (Sparks et al. 2005) and pear (Chitu & Paltineanu 2020). Many fruit trees go into a dormancy phase over the winter, a period of restricted growth that protects them from cold temperatures and frost damage (Campoy et al. 2011). A minimum amount of chilling time (a certain number of hours below a particular temperature), followed by forcing time (a certain number of hours above a particular temperature) is then required to stimulate vegetative growth and flowering (Guo et al., 2014). Chilling periods are often accumulated between October–December, whilst forcing times are accumulated from January–April (Drepper et al. 2020), although this can be location dependent.

Warmer forcing periods are likely to accelerate flowering due to faster heat accumulation (Ruiz et al. 2007), whilst warmer chilling periods can delay flowering due to insufficient chilling time (Guo et al., 2014). Reeves et al (2022) found that January–April temperatures had a significant effect on pear (*P. communis*) flowering time, with warmer temperatures associated with earlier flowering for 12 different pear cultivars and four phenological stages. Furthermore, this phenological advancement was predicted to continue, with full flowering becoming 18.5 days earlier under the highest emissions scenario (RCP 8.5) by 2080, providing chill requirements were met. Earlier budburst and flowering could have significant bottom-up impacts for this model system. Pear psylla nymphs often take shelter within rolled-leaves and flower buds, from natural enemies and adverse weather conditions (Reeves et al. 2022; Solomon et al. 1989), which could provide more protection for psyllids earlier in the year. In addition, adult females also increase oviposition rate when green foliage is present compared to dormant budwood (Horton 1990b); thus, if leaf flush is earlier, oviposition may also shift. With respect to spraying regimes, it is likely that pre-bloom sprays will need to shift, to account for earlier budburst, it is imperative that kaolin is applied pre-bloom to provide optimal spray coverage (Nottingham & Beers 2020). For anthocorid releases, this is dependent on how pest populations respond to earlier flowering. If psyllid oviposition and nymph emergence peaks earlier, then release of biological control should also shift, especially if natural anthocorid migrations do not follow this. This emphasises the importance of psyllid monitoring for growers, to optimally time sprays and mass releases.

Phenological monitoring is important within an agricultural ecosystem, allowing growers to decide when to apply different biological, chemical and cultural control methods (Fig. 3). A phenological model for *C. pyri* has been developed for the first and second-generation of pear psylla; this considers multiple variables including; termination of diapause, egg and nymph development, the pre-oviposition period and air temperature (Schaub et al. 2005). The model is now used in the SOPRA information system, for monitoring fruit pests in Switzerland, informing growers when to psylla are likely to emerge, when to monitor for them and the optimal time period to apply treatments (Samietz et al. 2007, 2011). However, this model has not been applied to UK regions, only considers the pest and looks air temperature rather than impacts of other weather variables. Thus, applying a pest forecasting system to UK pear orchards, which considers the phenology of pear, pear psylla and natural enemies with respect to weather variables would be optimal.

## Development and voltinism

Pear psylla and their natural enemies are poikilotherms (Reeves et al. 2023), meaning their body temperature fluctuates with their environment (Régnière & Powell 2013; Wojda 2017). Thus, the rate of development of poikilotherms is dependent on ambient temperature; developmental rate can also influence other variables such as voltinism (generations per year), fecundity and mortality (Culos & Tyson 2014). Insect development occurs between a critical thermal minima (CTmin) and a critical thermal maxima (CTmax) (Rebaudo & Rabhi 2018). Above CTmin development rate increases slowly with temperature at first, then linearly before it reaches an optimum (Topt). Once Topt is reached, there is a rapid decrease in development rate before the CTmax is reached. Temperature dependent development is evident in pear psylla (Kapatos & Stratopoulou1999); it is estimated that pear psylla have a CTmin of 10˚C for oviposition and egg development (Civolani 2012) and a CTmax of below 32.2 ˚C (McMullen & Jong 1977). However, the CTmax is based on *C. pyricola*, as the CTmax of *C. pyri* has not been recorded (Kapatos & Stratopoulou 1999; Schaub et al. 2005). Other authors have reported minimum temperatures that allow egg and nymphal development as 2–4 °C for *C. pyri* (Beránková & Kocourek 1994; Kapatos & Stratopoulou 1999; Schaub et al. 2005) and unsurprisingly changed with time of year due to temperature and changes in host quality (Civolani et al. 2023).

Studies predict that the number of generations per year is likely to increase in multivoltine insect species, due accelerated development resulting in the earlier completion of life cycles (Karuppaiah & Sujayanad 2012; Tobin et al. 2008). For *C. pyri*, the number of generations per year does differ spatially, likely due to climatic differences; with two generations per year recorded in Norway (Næss 2016), 3–4 generations in Switzerland (Daniel et al. 2005) and 5–6 generations in Greece (Stratopoulou & Kapatos 1992b). Furthermore, nymphs in Sicily overwinter alongside adults, as winters are far milder (Nin et al. 2012). Voltinism of *C. pyricola* also shows a substantial latitudinal gradient, with earlier maturation of eggs postdiapause and additional generations depending on latitude (Civolani et al. 2023). Thus, with UK summer temperatures predicted to increase (MetOffice 2022), elevated development rates could lead to an increased generation number. Differences in generational number have also been found for natural enemies of pear psylla; the multivoltine parasitoid *T. insidiosus*, completes 2–3 generations per year in France and Spain, whilst in Syria, six generations have been reported, due to higher temperatures in this region (Tougeron et al. 2021)*. A. nemoralis* also varies in generation number, with two generations in the UK (Solomon & Fitzgerald 1990), which can vary from 1 to 3 generations depending on location and host plant (Dempster 1963; Saulich & Musolin 2009).

Increased voltinism could have mixed effects for natural enemies depending on synchrony (Gaytán et al. 2022; Thomson et al. 2010), for parasitoids additional generations of hosts could provide a greater resource and increased time for population build-up (Horgan 2020). Alternatively, if host stage is asynchronous to the parasitoid, then there may be less hosts available to oviposit in or less time to complete its lifecycle. Furthermore, there is concern whether univoltine parasitoids and predators will have the plasticity to become multivoltine (Tougeron et al. 2020). Although there is evidence of multiple taxa shifting from univoltine to bivoltine lifecycles; for example, the spruce bark beetle *Ips typographus* is usually univoltine in Norway, Sweden and Finland; however, during warm summers, the species becomes bivoltine (Lange et al. 2006). Similar shifts have been found for the lawn ground cricket, *Polionemobius mikado*, which is bivoltine in southern Japan and univoltine in the north; however, this bivoltine lifecycle has slowly shifted northwards with respect to rising temperature (Matsuda et al. 2018).

## Fecundity, mortality and diapause

From late September onwards, winterform *C. pyri* adults begin to emerge (Bues et al. 1999). Winterform females are in reproductive diapause; where ovaries are still immature and experience a slow but constant development over the winter months (Lyoussoufi et al. 1994), whereas males have active sperm in the in the spermatheca (Civolani 2012; Hodkinson 2009). However, there is discussion whether rising temperatures will reduce the length of diapause (Karuppaiah & Sujayanad 2012; Kaur et al. 2023). For *C. pyri*, diapause is induced by short photoperiods in late summer, early autumn and low temperatures (Hodkinson 2009; Stratopoulou & Kapatos 1995; Tougeron et al. 2021). Studies show that young nymphs (L1-L3) reared under short-day length (LD 12:12) and low temperature (< 15 °C) produce diapausal winterform adults (Hodkinson 2009; Nguyen 1972). For the duration and termination of diapause, temperature becomes a more important environmental cue as diapause progresses. Hodkinson (2009) states that diapause is termination for *C. pyri* when exposed to temperatures above 25 °C, irrespective of photoperiod. However, the minimum temperature for diapause termination is dependent on location and photoperiod. Thus, it is likely that climate change could impact the duration of *C. pyri* diapause, with milder winter temperatures resulting in advanced emergence of adults from shelters and earlier egg laying (Civolani 2012). Multiple natural enemies of *C. pyri* enter diapause overwinter, including anthocorids (adults diapause under short-day conditions (Saulich & Musolin 2009), earwigs (enters a post-reproductive diapause under short photoperiods and low temperatures) (Goodacre 1998) and the multivoltine parasitoid *T. insidiosus* (Tougeron et al. 2021). For *T. insidiosus,* larvae overwinter inside *C. pyri* mummies; however, the photoperiodic or thermal cues required to induce this are unknown, highlighting an area of further research.

Mortality overwinter is particularly high for *C. pyri* adults, likely due to adverse weather conditions, limited resources and active winter predators (Horton et al. 1992; Kapatos & Stratopoulou 1996; Petráková et al. 2016). Kapatos & Stratopoulou (1996) found that on average, only 23.2% of *C. pyri* females survived overwinter, until the beginning of the oviposition period. Furthermore, rainfall and temperature have been shown to significantly impact psyllid mortality over winter (Horton et al. 1992; McMullen & Jong 1977), alongside habitat complexity (number of overwintering shelters) (Michalko et al. 2017) and predator abundance/activity (winter-active spiders such as *Anyphaena* and *Philodromus* can help to control psyllid populations) (Petráková et al. 2016); thus, milder winters could reduce psyllid mortality. In addition, temperature has a significant impact on summerform mortality, McMullen & Jong (1977) found that mortality rates of *C. pyricola* eggs and nymphs were lowest at 21.1 °C, with a higher longevity of summerform adults at lower temperatures compared to elevated temperatures. Furthermore, longevity under elevated temperatures significantly differed depending on morphotype, with summerform adults surviving significantly longer than winterforms (for temperatures > 30 °C). Higher temperatures also influenced fecundity in this study, with maximum fecundity at 21.1 °C (444.9 eggs per day), and significantly reduced oviposition rates at 35.0 °C (2.8 eggs per day). Once again optimum fecundity temperature depended on morphotype and was significantly lower for winterform females (15.6 °C). However, studies are lacking for *C. pyri* on fecundity and mortality, unlike the wide range of temperature regimes McMullen & Jong (1977) use *for C. pyricola*. Thus, it is difficult to confirm whether there are any temperature specific differences between *C. pyri* and *C. pyricola*. Further exploration of how RH impacts mortality and development is required, as young nymphs and eggs are vulnerable to desiccation under high temperatures and low humidity (Wilde 1964), suggesting these factors could interact synergistically.

## Feeding rates and functional responses

Climate change is predicted to have mixed effects on the feeding rates of sap-sucking insects (Evans & Borowicz 2015; Kenneth & Jayashankar 2020). Firstly, elevated CO_2_ levels could increase the C:N ratio in crops due to the fertilisation effect (Gifford 2004; González de Andrés, 2019); currently the rate of photosynthesis is limited as both CO_2_ and O_2_ compete for the active site of the rubisco enzyme used in photosynthesis. However, climate change may lead to higher levels of CO_2_ saturating rubisco’s active site; increasing amount of carbon fixation (McGrath & Lobell 2013). Thus, as nitrogen is already a limiting factor in the diet pear psylla (Le Goff et al. 2019; Pfeiffer & Burts 1984), the higher C:N ratio could result in increased compensatory feeding for phloem feeders to obtain essential amino acids (Ryan et al. 2010). Pfeiffer & Burts (1984) found that pear psylla had increased feeding rates and honeydew production on pear trees with lower nitrogen content, supporting this hypothesis. On the other hand, the upregulation of carbon-based chemical defence compounds may be enhanced under elevated CO_2_ (Robinson et al. 2012; Ryan et al. 2010). A meta-analysis by Robinson et al (2012) found a significant increase in tannins and overall leaf toughness under elevated CO_2_. However, increased leaf toughness may be more detrimental to folivores compared to phloem feeders; furthermore, trichrome hairs which provide a physical barrier for phloem feeders were not found to increase in density under elevated CO_2_, suggesting minimal impacts for sap-sucking insects with respect to plant defence.

Climate change may also alter transpiration rates of plants, depending on temperature, water stress, RH and CO_2_ level (Kirschbaum 2004; Mahato 2014). Furthermore, many factors interact synergistically (Reynolds-Henne et al. 2010; Schulze et al. 1973). Schulze et al (1973) found higher temperatures increased stomatal conductance; however, higher temperatures coupled with water stress significantly reduced stomatal conductance. Decreased transpiration rates can reduce plant vigour and accessibility to nutrients in the phloem for sap-sucking insects (Evans & Borowicz 2015). However, intermittent drought stress may be beneficial for phloem feeders, due to the pulsed stress hypothesis; where periods of stress, followed by the recovery of turgor, result in stress-induced increases in plant nitrogen (Huberty & Denno 2004). Therefore, it is important to consider interactions between weather variables, as well as their intensity and duration when predicting psyllid feeding rates with respect to climate change.

A functional response can be defined as the consumption rate of a predator depending on prey density (Holling 1965; Real 1977). It consists of attack rate; the rate at which a predator encounters a prey item and handling time; the time taken for a predator to consume the prey item (Juliano 2020; Real 1977). Functional responses are temperature dependent (Englund et al. 2011; Hassanzadeh-Avval et al. 2019); attack rates and handling times have been shown to vary with temperature in a hump-shaped manner (Uszko et al. 2017) and are often maximised at intermediate temperatures (Uiterwaal & DeLong 2020). Reeves et al (2022) demonstrated that anthocorid *A. nemoralis* did not significantly alter its attack rate or overall consumption rate of *C. pyri* nymphs depending on temperature, for current and predicted summer temperatures by 2080. However, this study concentrates on a small temperature range (18—23 °C) based on predicted UK average temperatures; for a larger temperature range significant differences may be evident. Hassanzadeh-Avval et al (2019) found significantly higher attack rates for *Anthocoris minki* Dohrn predating upon *Psyllopsis repens* Loginova at 30 °C compared to 15 °C, which may be relevant for maximum and minimum summer temperatures; however, these intervals have not tested for *A. nemoralis*. Temperature also interacts with other weather variables, impacting functional response; Yanik (2011) suggests that the combined effect of temperature and humidity had a significant impact on the consumption rate of *Ephestia kuehniella* Zeller eggs by *A. nemoralis*, whilst neither variable was significant alone.

## Behaviour, activity and spatial distribution

Dispersal of *C. pyri* winterform adults from orchards begins in September, peaking late October to early November (Civolani & Pasqualini 2003). The timing of this phenological event is dependent on temperature, humidity, precipitation and leaf fall (Horton et al. 1994). Civolani & Pasqualini (2003) showed that early *C. pyri* dispersal was correlated with early leaf fall and temperature. Thus, if leaf fall shifts with respect to climate change, psyllid dispersal may follow suit. Additionally, the field experiment highlighted that *A. nemoralis* sort refuge when maximum temperature dropped below 10 °C, demonstrating sheltering behaviour. Similar findings were seen for Coccinellidae spp. but for a higher maximum temperature. Furthermore, Horton et al (1994) demonstrated for *C. pyricola* that warmer and drier autumns lead to earlier dispersal and increased psyllid flight activity compared to those that were cool and wet.

The spatial distribution of pear psylla within the tree canopy impacts their activity and varies throughout the year (Horton 1994; Stratopoulou & Kapatos 1992a). Stratopoulou & Kapatos (1992a) monitored the spatial distribution of *C. pyri* within pear trees (eggs and young nymphs); their findings indicated that during spring psylla density was higher in the upper canopy, especially south or west facing; however, later in the year, numbers increased in the lower canopy. This could suggest that areas exposed to more sunlight were actively chosen as oviposition sites, to meet temperature requirements for development; however, later in the year, it may be more optimal to oviposit lower down in the canopy to reduce desiccation of eggs. Moreover, females displayed an oviposition preference for flowerbuds; 93.8% of eggs and nymphs were found in flowerbuds compared to leafbuds. This may be because it is more optimal for nymphs to develop inside flowers, as it provides more shelter from weather conditions and natural enemies (Reeves et al. 2022; Solomon et al. 1989). With respect to rising temperature, it is important to explore whether oviposition in the lower canopy increases during the summer, leading to spatial shifts in the psyllid population.

Spatial shifts in prey density under warming temperatures may lead to corresponding shifts for predators (Schmitz & Barton 2014). For example, climatic warming could lead to higher temperatures in the upper part of a plant canopy, prey respond by moving down to the lower canopy. Predators and parasitoids may also shift spatially due to rising temperature or to follow the distribution of prey (Barton & Schmitz 2009). For example, aphids often move downwards, occupying more shaded leaves in the lower canopy due to high summer temperatures (Dixon & Hopkins 2010). Aphid parasitoids also been shown to follow the distribution of aphids; a field-study monitoring pecan aphids found that the parasitoid *Aphelinus perpallidus* (Gahan) was most abundant in the lower canopy, where the population of pecan aphids were highest during the summer (Slusher et al. 2022).

However, when multiple predators are present the interactions can become more complex, with respect to climatic warming (Barton & Schmitz 2009; Schmitz & Barton 2014). Predators that usually occupy separate spatial niches within the plant canopy may overlap, leading to interference competition (when one predatory species reduces prey capture for a second predator species) or intraguild predation (IGP, where different predators consume each other, in addition to their target prey) (Jonsson et al. 2017). Therefore, it is important to identify natural enemies of *C. pyri* that could resort to IGP if niches overlap, as well as predicting spatial shifts of pest populations within the plant canopy. *F. auricularia* has a varied diet of insect, animal and plant material (Helsen et al. 1998); however, they are nocturnal (Suckling et al. 2006), so are less likely to interact with other natural enemy species. IGP has been documented between ladybird and lacewings (Karami-jamour et al. 2018; Zarei et al. 2020) and between *A. nemoralis* and multiple coccinellid species (Batuecas et al. 2022), indicating an avenue for further research.

## VOCs and trophic signalling

**Fig. 4:**
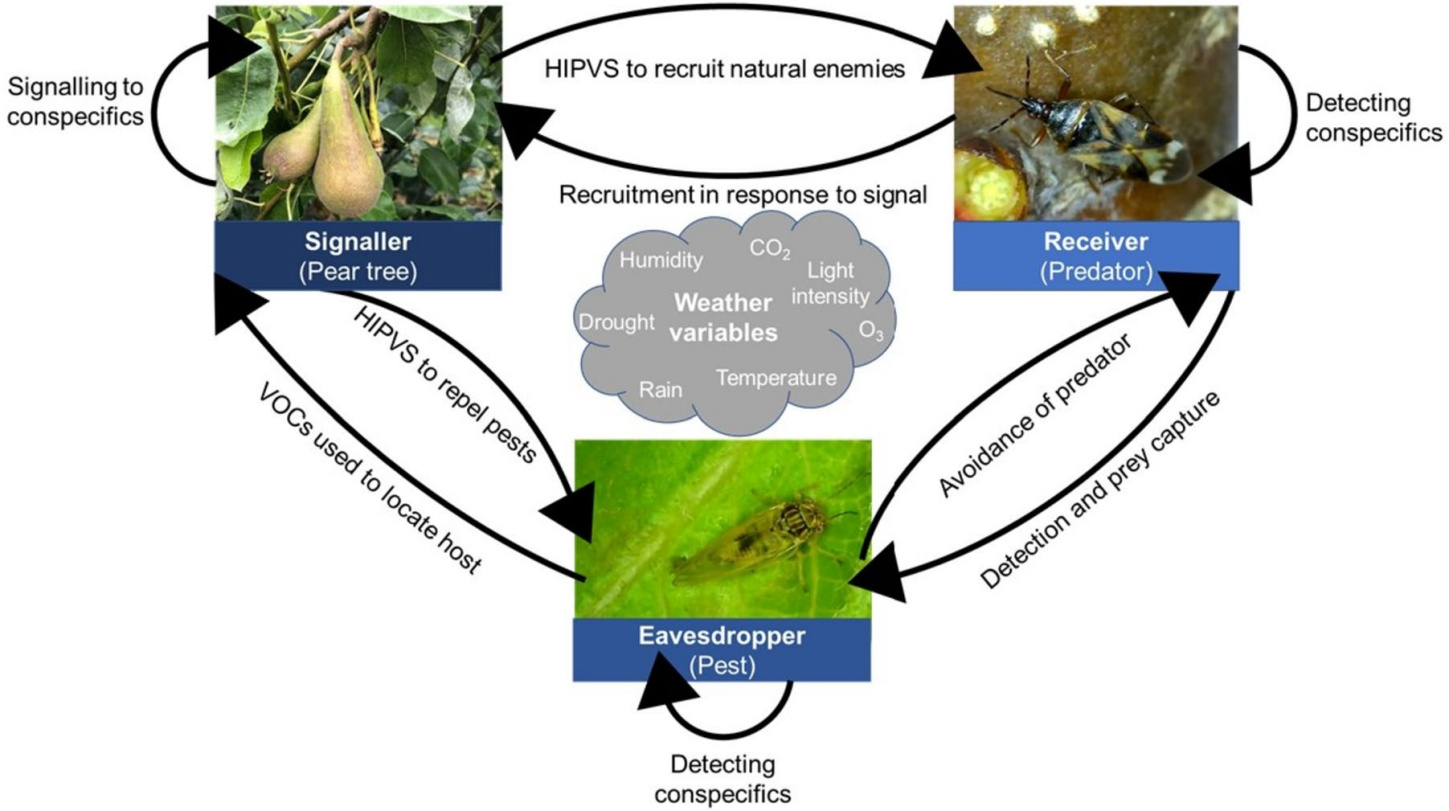
Signals and cues used within tri-trophic interactions between pears, pests and natural enemies, and the weather variables with the potential to alter or disrupt them. Cues and signals are used in a range of ways: HIPVs (herbivore-induced plant volatiles) can be used by plants to recruit predators and parasitoids and signal to conspecifics to upregulate genes for plant defence. However, plant VOCs (volatile organic compounds) can be eavesdropped upon by pests to detect hosts. Pheromones, acoustic and visual signals can be used to attract mates and detect conspecifics for insects. These signals can also be eavesdropped upon by natural enemies to locate prey

Pear psylla rely on a range of cues and signals resumed in Civolani et al. (2023); including chemical cues for host choice and oviposition (Gallinger et al. 2023; Horton & Krysan 1991), substrate-borne acoustic signals used in mate location (Eben et al. 2015; Jocson et al. 2023), tactile cues used when depositing eggs (Horton 1990a) and visual cues used to locate host plants (Adams et al. 1983; De Jorge et al. 2023). Abiotic factors have the ability to disrupt or alter cues and signals; acting as environmental noise, so it more difficult for the receiver to understand them (Lawson & Rands 2019; Lawson et al. 2017). Rainfall, temperature, light intensity, wind, humidity, CO_2_ and tropospheric ozone all have the ability to disrupt signals or create environmental noise (Lawson & Rands 2019; Yuan et al. 2016). Signal disruption may be further exacerbated by climate change (Fig. 4); via altered signal production, impacted transmission and changes in receiver perception (Becker et al. 2015; Yuan et al. 2009). Thus, it is vital to monitor how vulnerable pears, pear psyllid and their natural enemies are to signal disruption with respect to climate change.

One important set of infochemicals used in multitrophic communication is volatile organic compounds (VOCs) (Abbas et al. 2022; Yuan et al. 2009). In response to herbivory, plants often release herbivore-induced plant volatiles (HIPVs), which can recruit natural enemies (Allison & Daniel Hare 2009; Valle et al. 2023), repel pests (Turlings & Ton 2006) and can be used for plant–plant communication, resulting in increased upregulation of defence genes for receivers (Ninkovic et al. 2021). However, abiotic factors may influence VOCs; elevated temperature has been shown to alter the rate of transmission, emission and composition of VOCs (Helmig et al. 2007; Yuan et al. 2009). Isoprene is enhanced under climate warming and emission rates are positively correlated with temperature (Guenther et al. 1993; Loivamäki et al. 2008). A free-air carbon dioxide enrichment (FACE) experiment by Gallinger et al (2023) indicated that pear trees cultivated under elevated CO_2_ differed in their release of VOC compounds compared to ambient controls. Despite altered VOC emission, *C. pyri* females did not have a significant preference between trees grown in ambient or elevated CO_2_, in olfactometer or binary choice oviposition assays. However, whether the detection of HIPVs by natural enemies was altered, it was not investigated. This suggests an avenue of further research, especially as HIPVs can result in attractive responses for both anthocorids (Drukker et al. 2000; Scutareanu et al. 1997) and lacewing larvae (Valle et al. 2023). Climate change may also impact insect pheromonal communication; temperature has been shown to increase volatility and diffusion rates of semiochemicals, impacting transmission rate (Boullis et al., 2016). The pear psyllid pheromone is a long chain cuticular hydrocarbon (13-Me C27) with a low volatility, so the pheromone is likely to act at close range or is contact based (Civolani et al. 2023). Therefore, the impact on transmission rate may be less important, although further research on the relationship between 13-Me C27 and temperature is required.

Acoustic signals used for mate location and courtship can be temperature dependent (Larson et al. 2019; Yang et al. 2021; Jocson et al. 2023). Different components of acoustic signals can be thermally sensitive, including the pulse frequency, duration and interval between pulses (Larson et al. 2019; Walker & Cade 2003). An experiment by Jocson et al., (2023) demonstrated that song frequency of male pear psylla was temperature dependent, displaying a positive linear relationship with temperature (ranging from 180 to 1900 Hz). However, no significant relationship was found between pulse interval, pulse length and number of pulses and temperature. Whether higher frequency calls were more attractive to female psyllids, it was not assessed, making it unclear if temperature is likely to disrupt mating. On the other hand, rainfall is more pronounced in its disruption of acoustic communication, generating high-frequency vibrations of 3–4 kHz, acting as environmental noise for Homoptera (Tishechkin 2013). Psyllids usually cease to produce signals entirely in the presence of wind and rainfall to reduce energy consumption, in the generation of disrupted signals (Liao et al. 2022; Tishechkin 2013). Thus, alongside its ability to remove VOCs, increased rainfall can be disruptive to insect mating.

## Future outlooks

Pear psylla (*Cacopsylla pyri*) are a still a key pest of UK pear orchards, causing damage especially through the production of honeydew by nymphs, resulting in the growth of black sooty mould on shoots, foliage and fruit (Civolani et al. 2023). With the diminishing number of approved pesticides to control *C. pyri* and the resistance to previously used agrochemicals (Civolani et al. 2023), it is clear that biorational compounds, biological control and cultural control methods are being adopted by the UK pear growers, focusing on both top-down and bottom-up control. With application of the particle film kaolin and release of the biocontrol agent *A. nemoralis*, in several surveyed orchards (Table 1). It should be noted that multiple pesticides commonly used in pear psyllid management have been withdrawn over the past seven years (Hertfordshire 2023; HSE 2023), with a the withdrawal of a fourth compound (indoxacarb) currently planned for 2024. This review recommends applying a whole ecosystem approach to pear psyllid management that utilises regular pest monitoring, uses cultural and biological control methods and biorational compounds as alternatives to chemical sprays when possible and considers application timing depending on weather variables and phenological events.

The enhancement of natural enemies should be further encouraged by growers; *A. nemoralis* is a well-known natural enemy of *C. pyri*, currently mass released as a biocontrol agent in pear orchards (Sigsgaard et al. 2006a); however, other methods are recommended to enhance wild natural enemies populations (Shaw et al. 2021), rather than relying solely on mass released biocontrol. Refugia are key to cultural control methods within pear orchards to increase natural enemy populations within the tree canopy (Solomon et al. 1999). This includes artificial refuges such as corrugated cardboard in a bottle (Hansen et al. 2005; Solomon et al. 1999) or wooden Wignests loaded with food attractant (Russel-IPM 2023; Shaw et al. 2021) and natural refugia like native hedgerows (Nagy et al. 2008; Scutareanu et al. 1999), nettles (Shaw et al. 2021), cover crops (Horton et al. 2009) and wildflower strips or margins (Balzan et al. 2014; Mateos-Fierro et al. 2021). Furthermore, with the predicted surge in extreme weather events (MetOffice 2019), shelter for natural enemies may become increasingly important.

Exploration of rearing of other natural enemies aside from *A. nemoralis* is recommended; although *A. nemoralis* is likely to be an effective predator under predicted UK temperatures (Reeves et al. 2023), studies indicate that diverse predator assemblages can be more effective at controlling pest populations (Tylianakis & Romo 2010), providing that there is niche separation. Earwigs have a lower dispersal distance, so need to be released at multiple points in an orchard (Moerkens et al. 2010); however, they have good potential as biocontrol agents (Booth et al. 1992); thus, rearing and mass release within pear orchards should be further explored, alongside factors that influence their abundance within and between orchards. *Trechnites insidiosus* is a parasitoid wasp of interest, specific to pear psylla, with the ability to oviposit in all five nymphal instars (with a preference for third and fourth instars) (Le Goff et al. 2021; Tougeron et al. 2021). Tougeron et al (2021) proposed the release of *T. insidiosus* alongside other psyllid bicontrol agents during spring, although emphasises the need for further research into mass rearing to make the strategy cost-effective. However, there is a lack knowledge on the UK *T. insidiosus* populations, highlighting the need for parasitoid monitoring in the UK orchards.

The use of a combination of methods as an alternative to chemical insecticides is recommended to suppress pear psylla below economic thresholds (Shaw et al. 2021). Thus, the use and further development of biorational compounds and cultural control methods are advocated alongside biological control. In addition to kaolin, there are several methods currently absent from surveyed orchards that have potential for psyllid control. Firstly, oils can be an effective oviposition deterrent and repellent for *C. pyri* adults during the pre-bloom stage (Civolani 2012; Emami 2023; Erler 2004a, b). Effective oils include mineral (Civolani 2000), cotton seed, fish-liver, neem ( Erler 2004a, b) and peppermint oil (Li & Tian 2020), although some oils contain allergens making them unsuitable for the UK approval. Reflective plastic mulch is effective in psyllid population suppression (Nottingham & Beers 2020; Nottingham et al. 2022), reflecting solar light into the tree canopy (Shimoda & Honda 2013), promoting adult flight behaviour (Nottingham & Beers 2020) and damaging psylla eggs and nymphs (Beard 1972). However, there are concerns that elevated UV could impact natural enemies (Nottingham & Beers 2020), highlighting a need for further field trials. Plant defences elicitors are a potential approach to bottom-up control via activating plant defence pathways (Orpet et al. 2021; Saour et al. 2010; Civolani et al. 2022); however, studies have found variable pear psyllid suppression using PDEs (Cooper & Horton 2017; Orpet et al. 2021; Civolani et al. 2022), suggesting that PDEs should be used alongside other control methods. Finally, the discovery of a sex pheromone, produced by *C. pyri* females, is promising (Ganassi et al. 2018); this could be valuable as a pheromone lure for monitoring, trapping or mating disruption (Guédot et al. 2009). Acoustic signals also share this potential (Jocson 2023; Jocson et al. 2023); however, further field trials are required to evaluate their proficiency in mating disruption.

Climate change is likely to alter multiple processes within this agroecosystem; pear flowering phenology has advanced significantly over the past 60 years in the UK with respect to rising air temperature (Reeves et al. 2022), whilst insect pests and their natural enemies are poikilothermic (Régnière & Powell 2013; Wojda 2017); thus, development rate (Rebaudo & Rabhi 2018), voltinism (Karuppaiah & Sujayanad 2012), functional response (Englund et al. 2011; Hassanzadeh-Avval et al. 2019), mortality, oviposition (Culos & Tyson 2014) and even call frequency (Jocson 2023) can be temperature dependent. Furthermore, climatic warming can lead to spatial shifts in prey density (Schmitz & Barton 2014); predators can also shift their position within the plant canopy under higher temperatures, potentially resulting niche overlap, IGP and interference competition with other predator species (Barton & Schmitz 2009). A large proportion climate change-related studies focus solely on temperature, rather than other abiotic factors (Barton & Schmitz 2009; Clusella-Trullas et al. 2011; Kollberg et al. 2015). However, other abiotic factors such as precipitation, humidity, CO_2_ levels (Montoya & Raffaelli 2010), ozone, nutrient availability (Agathokleous et al. 2020; Yuan et al. 2009) and frost days (Sunley et al. 2006) should also be considered, as they can significantly impact trophic interactions and ecosystem services, with the potential to interact additively, synergistically or antagonistically.

Phenological mismatches are a particular concern for agroecosystems, as not all species respond equally to climate change (Damien & Tougeron 2019; Renner & Zohner 2018). Although phenological models have been created for pear psylla and natural enemies, they are often look at the organism in isolation rather than its interaction with other trophic levels (Moerkens et al. 2011; Schaub et al. 2005). These interactions could be particularly important, for example how pear budburst corresponds with pear psyllid oviposition or how anthocorid migration into pear orchards depends on psyllid population density, making it imperative to consider primary producers, pests and natural enemies when creating phenological models, as the shifting of one level could create mismatches for others. An App to record phenological monitoring data for multiple trophic levels (pear tree, pear psylla and natural enemies) would be beneficial for UK pear growers, allowing the input of data and guidance of when to apply certain control methods based on phenological stage and pest abundance. It would also provide data for researchers, allowing them to link key phenological events to weather variables and help model pear psyllid populations, for a year-on-year basis and under future climate scenarios.

## Conclusion

This review proposes a whole ecosystem-based approach for pear psyllid management; that considers cultural, biological and chemical control methods, application timing, habitat management and abiotic processes that may disrupt pest management. There are a diverse range of methods currently used to control pear psylla. However, with the reduction in insecticides approved for the UK use and the potential disruption to trophic interactions as a result of climate change, the timing of these control methods may need to shift or alternative methods may need to be applied. Climate change has the potential to alter both bottom-up and top-down processes within ecosystems. Abiotic factors such as temperature, humidity, rainfall, drought, light intensity, ozone and CO_2_ could impact bottom-up control by affecting nutrient uptake, availability and plant defence, as well as top-down control impacting predator activity, IGP, interference competition and functional responses. Changes in phenology, feeding, oviposition and activity are all important factors that must be monitored in respect to climate change to inform effective and timely interventions. For monitoring tri-trophic interactions, signalling responses should be considered, including VOCs and pheromones for chemical signalling, tactile signals herbivores use for oviposition, acoustic and visual signals used to attract mates and gustatory cues to differentiate between hosts and non-hosts. The need for phenological data in monitoring trophic interactions is vital, few growers and agronomists regularly monitor their orchards and record this information. These data could be used to help make decisions on spray timing or natural enemy release, as well as inform phenological models that predict pest populations and natural enemy emergence based on weather variables. Thus, an easily accessible App and collective database is recommended for the UK pest monitoring and control in pear orchards.

## Author contributions

All authors conceived and designed the review. LR wrote the manuscript, created the figures and analysed the spray records and pest monitoring data. All authors read and provided feedback on multiple drafts prior to submission.

## Data Availability

Dataset accessible from the University of Reading Research Data Archive.
